# Cross-matching: a modified cross-correlation underlying threshold energy model and match-based depth perception

**DOI:** 10.3389/fncom.2014.00127

**Published:** 2014-10-15

**Authors:** Takahiro Doi, Ichiro Fujita

**Affiliations:** ^1^Laboratory for Cognitive Neuroscience, Center for Information and Neural Networks, Graduate School of Frontier Biosciences, Osaka UniversitySuita, Japan; ^2^Center for Information and Neural Networks, National Institute of Information and Communications TechnologySuita, Japan

**Keywords:** binocular disparity, stereo vision, correspondence problem, random-dot stereogram, anticorrelated, nonlinearity, discrimination

## Abstract

Three-dimensional visual perception requires correct matching of images projected to the left and right eyes. The matching process is faced with an ambiguity: part of one eye's image can be matched to multiple parts of the other eye's image. This stereo correspondence problem is complicated for random-dot stereograms (RDSs), because dots with an identical appearance produce numerous potential matches. Despite such complexity, human subjects can perceive a coherent depth structure. A coherent solution to the correspondence problem does not exist for anticorrelated RDSs (aRDSs), in which luminance contrast is reversed in one eye. Neurons in the visual cortex reduce disparity selectivity for aRDSs progressively along the visual processing hierarchy. A disparity-energy model followed by threshold nonlinearity (threshold energy model) can account for this reduction, providing a possible mechanism for the neural matching process. However, the essential computation underlying the threshold energy model is not clear. Here, we propose that a nonlinear modification of cross-correlation, which we term “cross-matching,” represents the essence of the threshold energy model. We placed half-wave rectification within the cross-correlation of the left-eye and right-eye images. The disparity tuning derived from cross-matching was attenuated for aRDSs. We simulated a psychometric curve as a function of graded anticorrelation (graded mixture of aRDS and normal RDS); this simulated curve reproduced the match-based psychometric function observed in human near/far discrimination. The dot density was 25% for both simulation and observation. We predicted that as the dot density increased, the performance for aRDSs should decrease below chance (i.e., reversed depth), and the level of anticorrelation that nullifies depth perception should also decrease. We suggest that cross-matching serves as a simple computation underlying the match-based disparity signals in stereoscopic depth perception.

## Introduction

The stereoscopic system gives rise to three-dimensional visual perception by combining the images from the left and right eyes. To successfully combine the two images, the system needs to match a given part of one eye's image to the correct counterpart in the other eye's image projected from the same object. The positional difference between the correctly matched parts, called binocular disparity, is a quantitative depth cue for the stereoscopic system.

**Figure 1:**
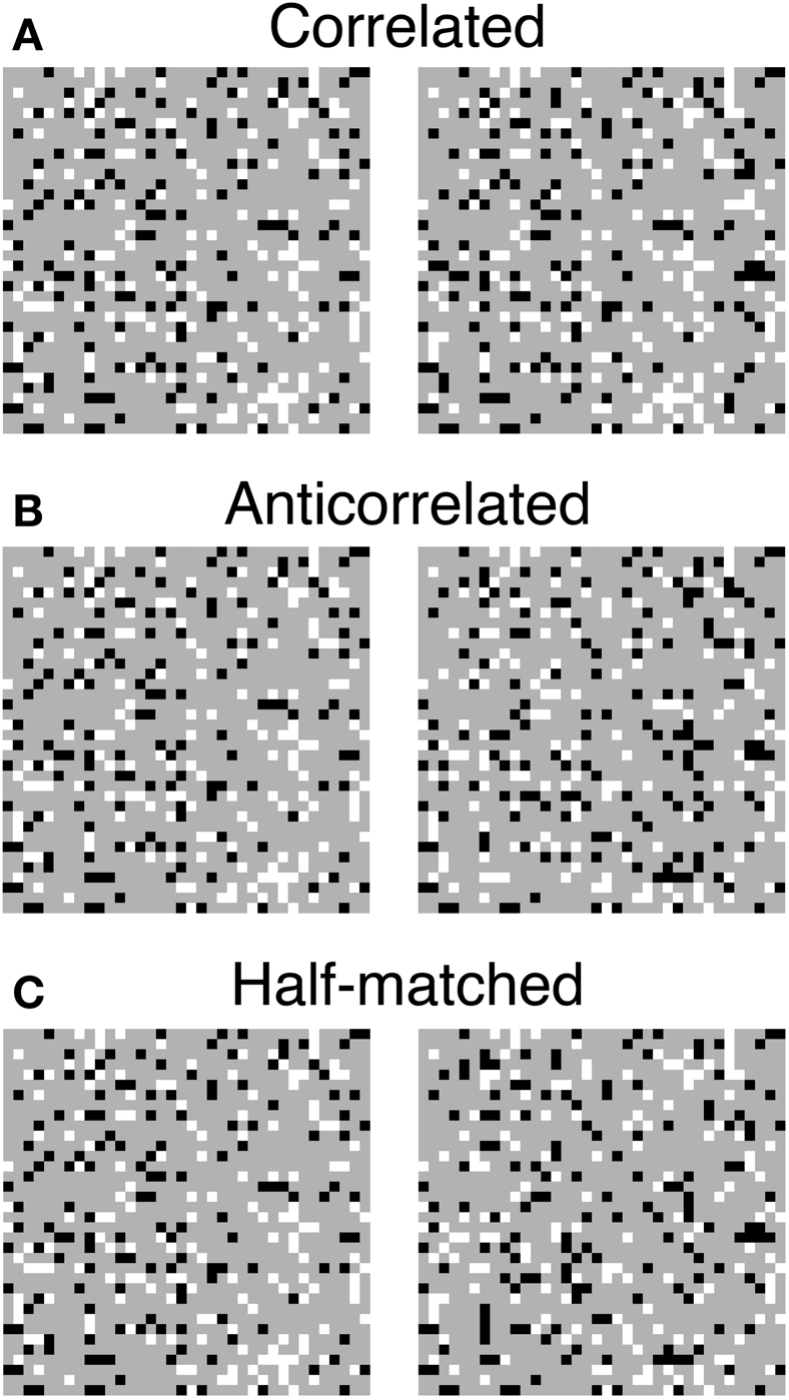
**Three random-dot stereograms (RDSs) with different binocular correlations. (A)** Correlated RDSs. Binocular fusion of the left and right half-images with parallel (uncrossed) viewing produces a sensation of a central square floating from background. **(B)** Anticorrelated RDSs. The luminance contrasts are reversed for all the dots in the central square of the right half-image. Reversed, correct, or no depth is perceived, depending on stimulus configuration. **(C)** Half-matched RDSs. Half of the dots in the central square of the right half-image have reversed contrasts. Correct depth can be perceived, since half of the dots carry the same disparity signals as in the correlated RDSs.

**Figure 2:**
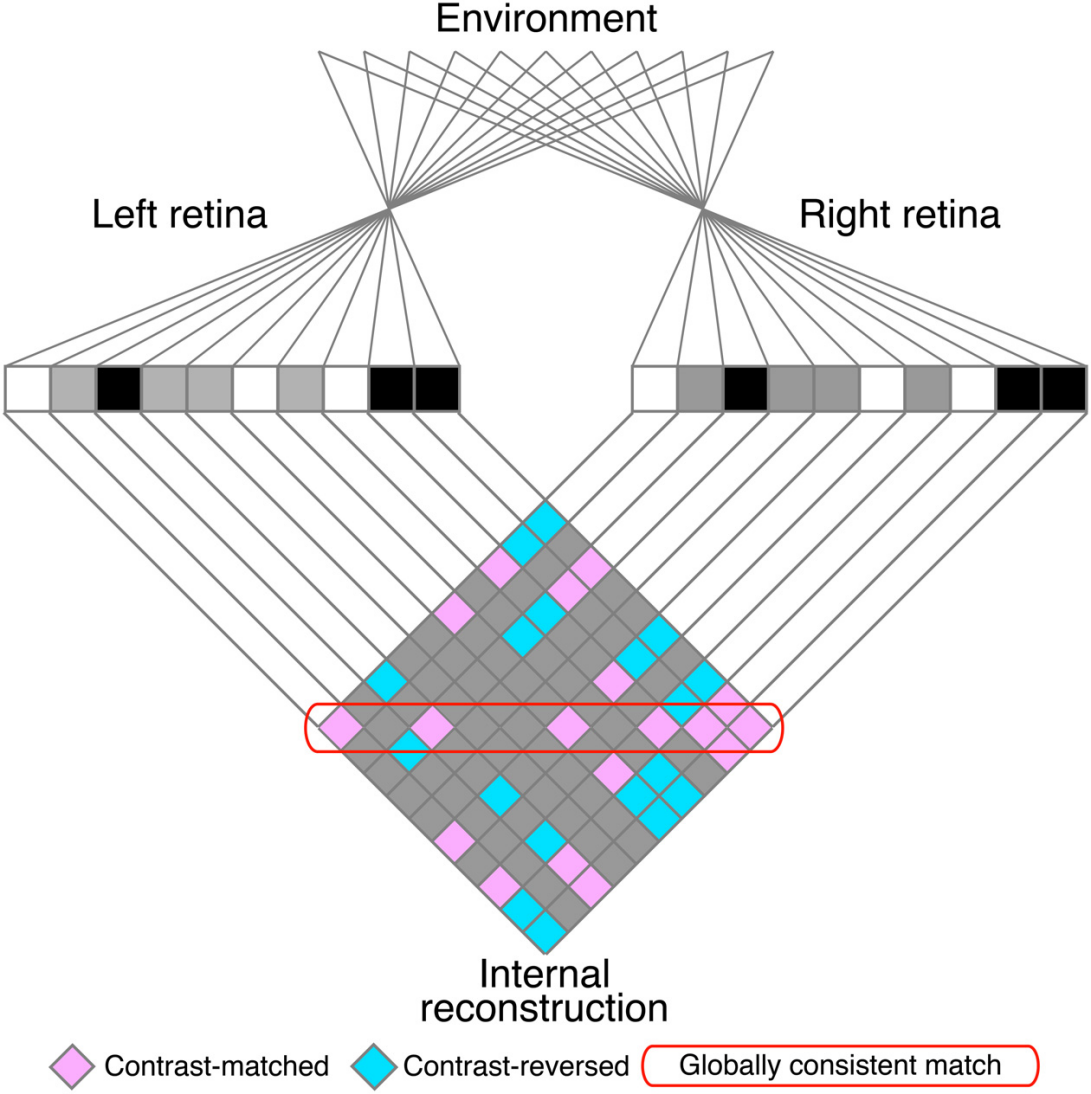
**A schematic illustration of the stereo correspondence problem**. The problem is illustrated using an example of one-dimensional random-dot stereograms. The visual system infers the three-dimensional structure in the environment **(top)** from the images projected to the left and right retinae **(middle)**. The process of solving the problem can be defined as finding the globally consistent solution (red rounded box) while discarding local matches that do not belong to the global solution (false matches, pink elements outside of the red rounded box). Aquamarine elements indicate contrast-reversed combinations. Gray elements indicate background–background or background–dot combinations.

The matching process is often confronted with the stereo correspondence problem, in which locally correct but globally incoherent matches (i.e., false matches) result in ambiguous solutions to the problem. The stereoscopic system must select correct matches and discard false matches in order to generate an appropriate representation of three-dimensional world (Julesz, 1971; Marr and Poggio, 1979). The correspondence problem is particularly complex for random-dot stereograms (RDSs; Figure 1; Julesz, 1971), in which identical black and white dots yield numerous false matches (Figure 2). Despite this complexity, human subjects can perceive a coherent three-dimensional structure embedded in RDSs, suggesting that the stereoscopic system is capable of selecting a globally consistent solution to the correspondence problem (red rounded box, Figure 2).

Anticorrelated RDSs (Figure 1B) are often used to test the neural representation of the globally consistent solution (Cumming and Parker, 1997; Nieder and Wagner, 2001; Janssen et al., 2003; Krug et al., 2004; Tanabe et al., 2004; Kumano et al., 2008; Preston et al., 2008; Theys et al., 2012). In anticorrelated RDSs, one eye's image is contrast-reversed to produce the photographic negative of the other eye's image. For this stimulus, the correspondence problem does not have a globally consistent solution. Therefore, neurons representing the solution should lose or reduce disparity selectivity for anticorrelated RDSs, while retaining sensitivity for correlated RDSs. Some neurons in the visual cortex, particularly in higher areas in the cortical hierarchy, exhibit such response properties (Janssen et al., 2003; Tanabe et al., 2004; Haefner and Cumming, 2008; Kumano et al., 2008; Theys et al., 2012).

Threshold nonlinearity after a disparity energy model explains the reduced disparity selectivity for anticorrelated RDSs (Lippert and Wagner, 2001; Nieder and Wagner, 2001). We call this model the “threshold energy model.” The threshold nonlinearity provides a sufficient explanation for neurons with even-symmetric tuning curves, and a further combination of energy-model subunits explains the reduced selectivity in odd-symmetric tuning curves (Haefner and Cumming, 2008; Tanabe and Cumming, 2008).

Cross-correlation of left- and right-eye images is the fundamental computation underlying the disparity selectivity of energy models (Fleet et al., 1996; Anzai et al., 1999; Filippini and Banks, 2009). Similarly, thresholded cross-correlation may represent the fundamental computation underlying the disparity selectivity of the threshold energy model. However, the characteristics of such a nonlinear extension of cross-correlation have yet to be examined in detail. In particular, the size of a spatial window before threshold nonlinearity should influence the output of thresholded cross-correlation. Here, we derived the analytical solution for the disparity signal of thresholded cross-correlation, for which we coined the term “cross-matching.” We examined two versions of cross-matching in responses to RDSs with graded anticorrelation (i.e., a graded mixture of anticorrelated and correlated dots). First, we considered the simplest version of cross-matching, in which the threshold operates with single-pixel resolution. Second, we explored a more general version of cross-matching, in which signals are spatially averaged with various window sizes prior to the threshold. We showed that both versions of cross-matching reproduced a nearly flat disparity-tuning function to anticorrelated RDSs. The first version also explained the match-based psychometric curve of near/far discrimination hypothesized and observed in our previous psychophysical studies (Doi et al., 2011). The results obtained with the first version of cross-matching were preserved in the second version, up to a modest extent of spatial averaging prior to the threshold. Finally, we derived testable predictions regarding how the match-based psychometric function should change if the dot density in RDSs is manipulated.

## Results

### Cross-correlation and cross-matching for correlated, anticorrelated, and half-matched RDSs

We examined cross-correlation and cross-matching when the following conditions were met. First, dot patterns of RDSs were updated over time while a three-dimensional structure defined by binocular disparity remained fixed (i.e., dynamic RDSs). Thus, responses to individual dot patterns could be averaged out. Second, a flat disparity plane was embedded in the RDSs (Figure 1). Third, the shape and position of the embedded disparity plane were given *a priori*, so that the spatial windows (receptive fields) could have matching shapes and positions. Because these conditions are often met in measurements of neuronal disparity tunings, our conclusions are applicable to a wide range of neurophysiological experiments.

**Figure 3:**
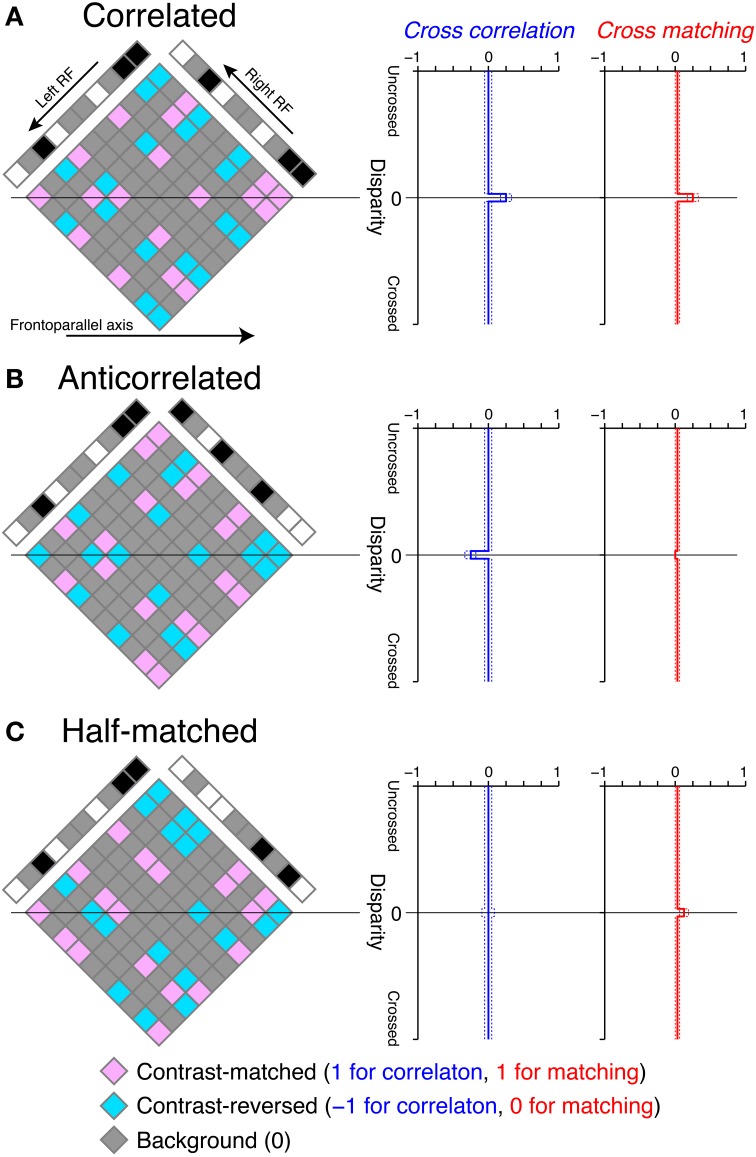
**Graphical illustration of cross-correlation and cross-matching for the detection of disparity in RDSs**. **(A)** Left, an example correlated RDS and the internal reconstruction (diamond). Right, expected cross-correlation and cross-matching as a function of receptive-field disparity (Equations 1 and 3, respectively, with a dot density of 25%). Dotted lines indicate the 95% intervals of the probability density functions, calculated with a pixel number of 1024. Our cross-correlation and cross-matching correspond to the spatial average of contrast-matched (pink) and contrast-reversed (aquamarine) elements in the diamond along the frontoparallel axis, with the codes described at the bottom of the figure. The temporal average across different random-dot patterns gives the smooth expected functions plotted at the right side. **(B,C)** Anticorrelated and half-matched RDSs, respectively, in the same format as in **(A)**.

Under these conditions, cross-correlation unambiguously signals the disparity embedded in correlated RDSs (Figure 3A). We used the cross-correlation (*C*) with the following form:

(1) $$C(d)=\langle \frac{1}{k}\sum {}_{\left(x,y\right)\in w}I_{L}\left(x,\;y\right)I_{R}\left(x-d,\;y\right)\rangle ,$$

where *d* indicates the disparity between the left-eye window (*W*) and the right-eye window (receptive-field disparity); *I_L_* and *I_R_* indicate the luminance contrasts of left-eye and right-eye images, respectively (1 for bright dots, 0 for background, and −1 for dark dots), as a function of horizontal (*x*) and vertical (*y*) positions; *k* indicates the number of elements (pixels) in the spatial window; and 〈·〉 indicates the expected value across an infinite number of time frames (i.e., different dot patterns).

We illustrated the cross-correlation by showing the horizontal sections of RDSs (Figure 3). The cross-correlation is the spatial average of possible binocular combinations (diamond field in Figure 3) along the horizontal (frontoparallel) dimension. The binocular combinations of contrast signals have the following codes: ***b*** = (*b_m_, b_r_, b_b_*) = (1, −1, 0), where *b_m_*, *b_r_*, and *b_b_* indicate the codes for contrast-matched (pink elements in Figure 3), contrast-reversed (aquamarine elements), and background–background or background–dot combinations (gray elements), respectively. We calculated the expected value of the cross-correlation by ***bp***^*T*^, where ***p*** = (*p_m_, p_r_, p_b_*) indicates the probability for a given binocular combination to be contrast-matched, contrast-reversed, and background, respectively. If the receptive-field disparity (*d*) matches the stimulus disparity (*d_s_*) (e.g., zero in the examples in Figure 3), $p=\left(\frac{\text{1+c}}{2}\rho ,\frac{\text{1}-c}{\text{2}}\rho ,1-\rho \right)$, where *c* and ρ indicate binocular correlation and dot density in units of probability, respectively. If *d* ≠ *d_s_*, $p=\left(\frac{\rho^{\text{2}}}{2},\frac{\rho^{\text{2}}}{\text{2}},\;1-\rho^{\text{2}}\right)$. Thus, the expected value of the cross-correlation *C* (*d*) = *c*ρ if *d* = *d_s_*, and 0 otherwise.

We also calculated the probability density function of the cross-correlation to examine the size of the variability originating from the randomness of the RDSs. The probability for the spatial window *W* to contain a given combination of contrast-matched, contrast-reversed, and background pixels is defined by the trinomial distribution with the following form:

(2) $$D(n,p)=\frac{k!}{n_{m}!n_{r}!n_{b}!}p_{m}^{n_{m}}\times p_{r}^{n_{r}}\times p_{b}^{n_{b}},$$

where ***n*** = (*n_m_, n_r_, n_b_*) indicates the numbers of contrast-matched, contrast-reversed, and background pixels, respectively, within the spatial window. The value of the cross-correlation is $\frac{1}{k}bn^{T}=\frac{n_{m}-n_{r}}{k}$ for a given ***n***. We constructed the probability density function of the cross-correlation across all possible combinations of (*n_m_, n_r_, n_b_*) that satisfy *n_m_* + *n_r_* + *n_b_* = *k*. In Figure 3, we showed the 95% interval of the probability density function calculated with *k* = 1024. With this window size and 25% dot density, the total number of dots becomes similar to that used in our previous psychophysical experiments (Doi et al., 2011, 2013).

For correlated RDSs, the cross-correlation has a peak at zero disparity, corresponding to the depth of the globally consistent solution (Figure 3A, blue). This is because all non-background binocular combinations are contrast-matched at the stimulus disparity, whereas contrast-matched and contrast-reversed combinations occur with equal probability and on average cancel each other out at other disparities in correlated RDSs. Cross-correlation (Equation 1) is most closely related to the disparity tuning of tuned-excitatory energy models. These neurons have receptive fields shifted in position between the two eyes, just as the cross-correlation has windows shifted in position. Neuronal disparity tuning is a function of stimulus disparity, and the cross-correlation is a function of window (receptive field) disparity. Both neuronal disparity tuning and cross-correlation peak when stimulus disparity and receptive-field disparity are aligned, but fall to baseline levels when the two disparities are different. For a neuronal tuning curve, the transition from peak to baseline is gradual because of the band-pass nature of receptive fields (Qian and Zhu, 1997).

We defined cross-matching as a nonlinear modification of cross-correlation with the following form:

(3) $$M(d)=\langle \frac{1}{k}\sum {}_{\left(x,y\right)\in w}{\left[I_{L}\left(x,\;y\right)I_{R}\left(x\;-\;d,\;y\right)\right]}^{+}\rangle ,$$

where [·]^+^ indicates half-wave rectification. In this version of cross-matching, we placed the half-wave rectification inside the cross-correlation, so that the threshold nonlinearity operated with single-pixel resolution. The cross-matching can be understood as easily as the cross-correlation, because the only difference is the code for contrast-reversed combination (−1 for the cross-correlation and 0 for the cross-matching). If we substitute the binocular-combination codes ***b*** with the new codes ***b′* = [*b*]^+^** = (1, 0, 0), the expected value of the cross-matching is ***b′ p***^*T*^ = $=\frac{1+c}{2}\rho$ if *d* = *d_s_*, and $\frac{\rho^{2}}{2}$ otherwise. Thus, the disparity tuning of the cross-matching is similar to that of the cross-correlation for correlated RDSs (Figure 3A), although the baseline height is slightly different: 0 for the cross-correlation but $\frac{\rho^{2}}{2}$ for the cross-matching (ρ = 0.25 for the examples in Figure 3, right).

Outputs of cross-correlation and cross-matching differed more prominently when RDSs were anticorrelated (Figure 3B). The cross-correlation became inverted, because in anticorrelated RDSs all binocular combinations at the stimulus disparity are contrast-reversed. However, the cross-matching was nearly flat for a low dot density (e.g., 25%), because half-wave rectification converts negative, contrast-reversed combinations to zero. The expected value is exactly zero when stimulus and window disparities are aligned, and it is close to zero otherwise. Thus, a simple nonlinear modification of cross-correlation is sufficient to explain nearly flat disparity tuning, at least for low-density RDSs (see below for high-density RDSs).

We found the opposite pattern of difference for half-matched RDSs, in which half of the dots were contrast-matched and the other half contrast-reversed (Figure 1C). Any purely cross-correlation-based mechanisms would be unable to detect the disparity of half-matched RDSs, because the signals between contrast-matched and contrast-reversed binocular combinations cancel each other out (see the zero-disparity line in Figure 3C). However, human subjects can perform a near/far discrimination task for half-matched RDSs, suggesting an involvement of separate match-based disparity detectors (Doi et al., 2011, 2013). Here, the cross-matching function serves as a computation underlying the match-based detector. As a result of half-wave rectification (Equation 3), contrast-reversed and contrast-matched binocular combinations do not cancel out by the spatial average. The expected peak height is generally higher than the expected baseline level. Thus, unlike the cross-correlation, cross-matching can signal the disparity embedded in half-matched RDSs.

### A full profile of signal strength as a function of binocular correlation and dot density

We next examined a more complete profile of cross-correlation and cross-matching by varying the dot density from 0 to 100%. In the examples above, we used a dot density of 25%, which has been often used in physiological and psychophysical studies with anticorrelated RDSs (Cumming and Parker, 1997; Krug et al., 2004; Tanabe et al., 2004; Kumano et al., 2008; Doi et al., 2011, 2013). We defined signed signal strength as the peak minus baseline of the cross-correlation or cross-matching. For cross-correlation, the expected signal strength (*S_C_*) has the following form:

(4) $$\begin{array}{l} S_{C}\left(c,\rho \right)=C\left(d=d_{s}\right)-C\left(d\neq d_{s}\right)\\ \;=c\rho \end{array}$$

**Figure 4:**
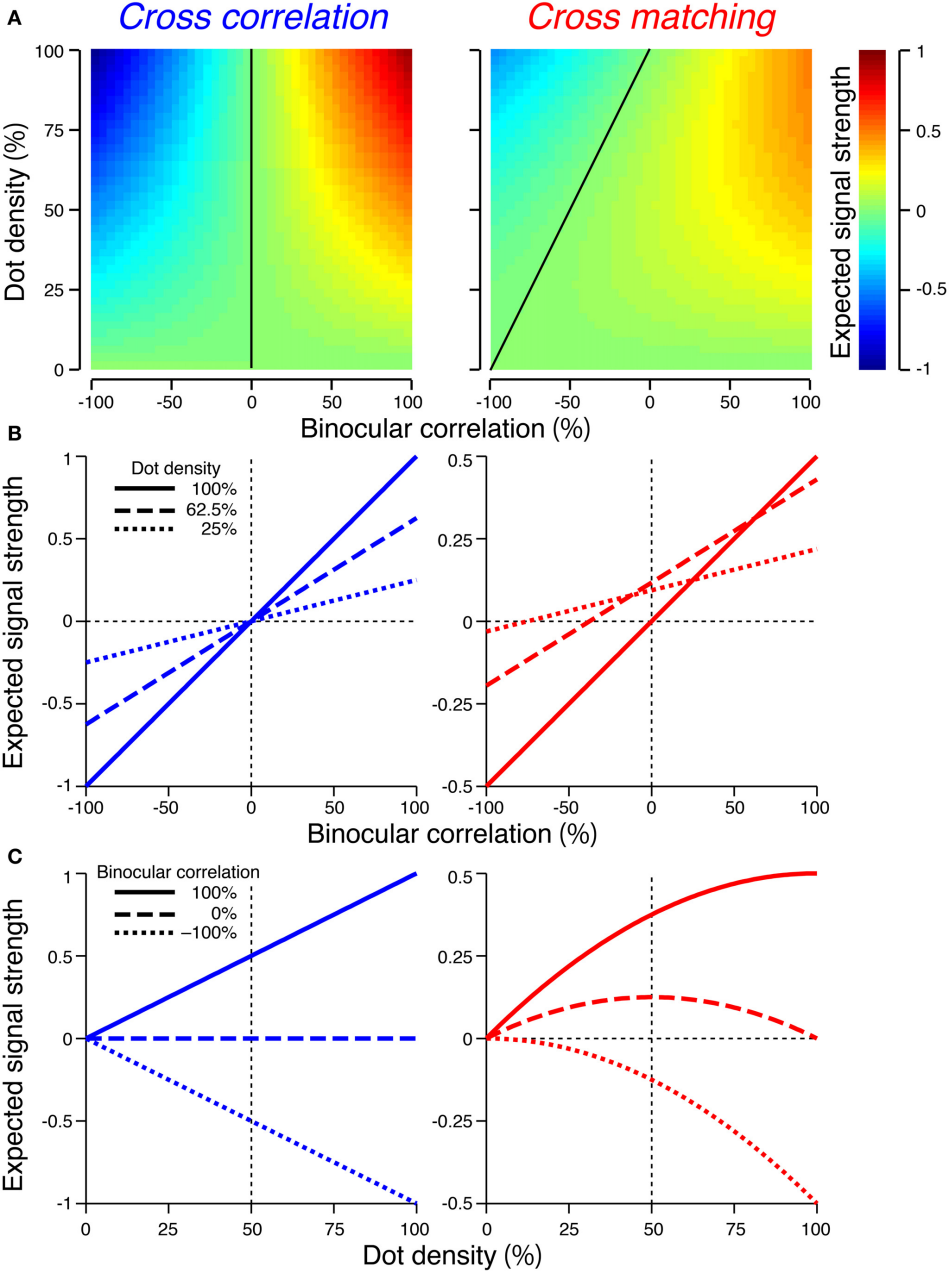
**Expected signal strengths of cross-correlation (left column) and cross-matching (right column)**. **(A)** Expected signal strength (peak − baseline) is plotted as a function of dot density and binocular correlation. Black line indicates zero signal strength. **(B)** Horizontal cross-section of the signal strength in **(A)**, as a function of binocular correlation. Solid, broken, and dotted lines indicate sections at 100, 62.5, and 25% dot densities, respectively. **(C)** Vertical cross-sections of the signal strength in **(A)**, as a function of dot density. Solid, broken, and dotted lines indicate sections at 100, 0, and −100% correlations, respectively.

The signal strength is separable into correlation and density terms, odd-symmetric relative to zero binocular correlation (Figure 4A, left, vertical solid line), and linearly dependent on both binocular correlation (Figure 4B, left) and dot density (Figure 4C, left).

For cross-matching, the expected signal strength (*S_M_*) has the following form:

(5) $$\begin{array}{l} S_{M}\left(c,\rho \right)=M\left(d=d_{s}\right)-M\left(d\neq d_{s}\right)\\ \;=\frac{\left(c+1\right)\rho }{2}-\frac{\rho^{2}}{2} \end{array}$$

The signal strength is not separable into correlation and density terms (Figure 4A, right). The zero-signal line slanted in the correlation-density map (Figure 4A, right, solid line). In other words, the signal strength, as a function of correlation, crossed the zero level at larger correlations for larger densities (Figure 4B, right). Notably, for a dot density of 100%, the profile was similar to that of cross-correlation (compare solid lines between Figure 4B left and right). However, as the dot density decreased, the signal strength changed nonlinearly in a manner dependent upon binocular correlation (Figure 4C, right). The positive signals near 100% correlation slowly decayed (Figure 4C, right, solid line), but the negative signals near −100% correlation diminished more quickly (Figure 4C, right, dotted line). At 0% correlation, the signal strength had a concave profile with a peak at 50% density (Figure 4C, right, broken line).

For anticorrelated RDSs, the peak of the cross-matching is always zero, whereas the baseline increases with the dot density. Thus, the signal strength becomes more and more negative with increasing density. For half-matched RDSs, the peak and baseline have the same values at 0% and 100% density; thus, the signal strength is zero at the two ends. Indeed, a half-matched RDS with 100% density is an uncorrelated RDS devoid of any form of disparity information. For intermediate densities, the peak is higher than the baseline, with the maximum difference at 50% density.

We also examined the variability of signal strength caused by the randomness of the RDSs, because the variability is relevant for psychophysical performance. To this end, we generated 1000 random-dot patterns for a given combination of dot density and binocular correlation, and calculated the standard deviation of the signal strength for the cross-correlation and cross-matching. All simulations in this study were performed using Matlab (Mathworks). An RDS consisted of a central square and a surrounding background, as in Figure 1. The central square had a size of 32 pixels × 32 pixels (i.e., 1024 pixels), whereas the surrounding area had a size of 34 pixels × 34 pixels. The disparity of the central square was −2 pixels (crossed disparity). The signal strength was simulated as the response difference between near-preferring and far-preferring units [*C* (*d* = −2) − *C* (*d* = 2) and *M* (*d* = −2) − *M* (*d* = 2) for the cross-correlation and cross-matching, respectively; note that here *C* (*d*) and *M* (*d*) indicate responses to an individual dot pattern, but not an expected value across different patterns]. The receptive-field size and location were adjusted to the center square of the RDS (*k* = 1024). We calculated the average standard deviation across 200 simulation runs.

**Figure 5:**
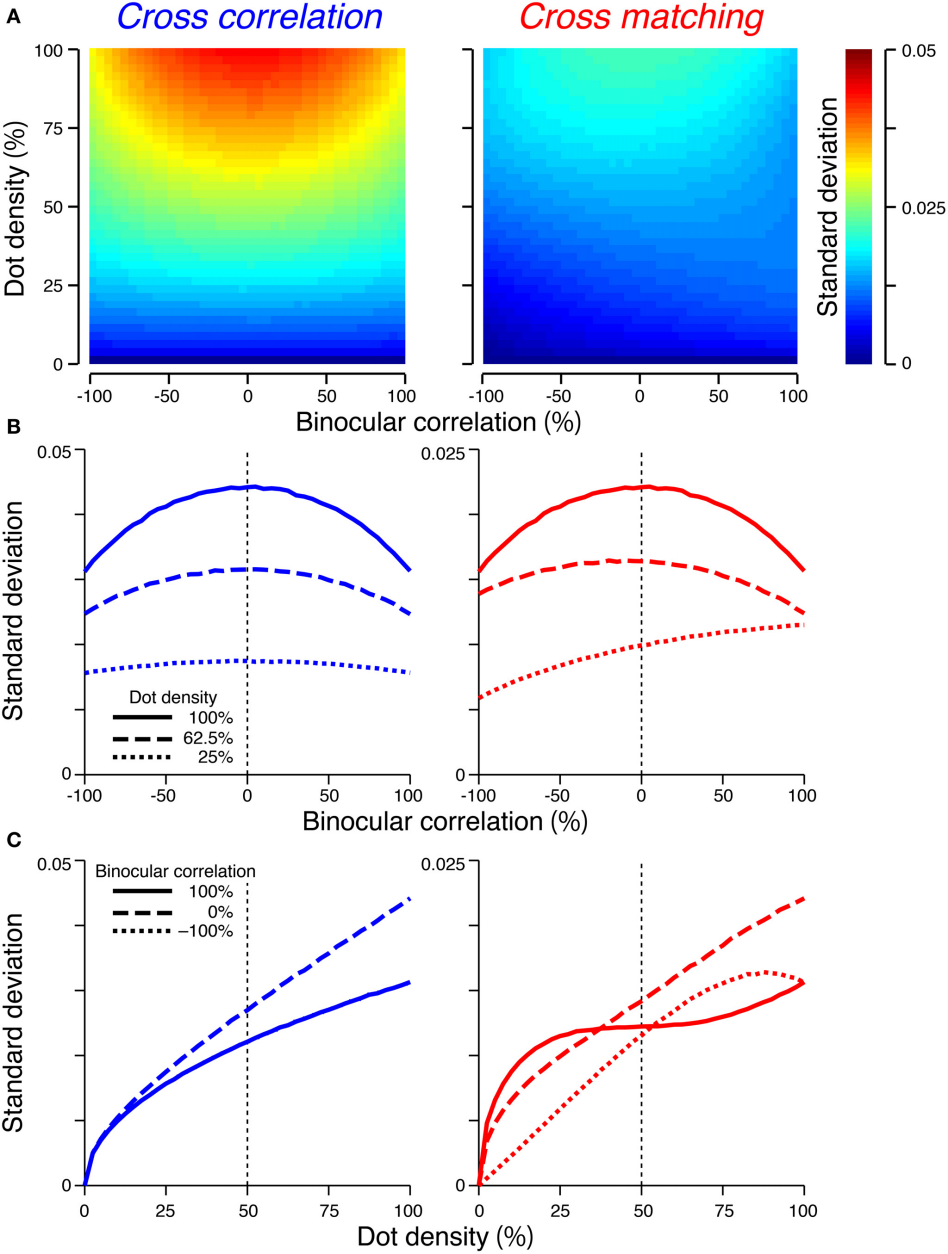
**Standard deviations of the signal strengths**. The figure convention is the same as that used in Figure 4, except for the color scale of **(A)** and the y-axis range of **(B,C)**. **(C)** The dotted line overlaps the solid line in the left panel.

When the number of pixel was sufficiently large (i.e., 1024), the scale of the standard deviation was smaller than that of the expected value. The maximum standard deviation was 0.044 and 0.022 for the cross-correlation and cross-matching, respectively (Figure 5). By contrast, the expected signal strength varies from 1 to −1 for the cross-correlation and from 0.5 to −0.5 for the cross-matching (Figure 4). Thus, when the number of pixels is matched to our experimental condition (Doi et al., 2011), the variability of the signal strength should have only a negligible effect on psychometric functions.

If the size of the spatial window is decreased, the overall scale of the standard deviation should increase, although the dependence of the standard deviation on binocular correlation and dot density (Figure 5) should be invariant with respect to window size. When the expected value and standard deviation have comparable scales, both should influence the shapes of psychometric functions.

### Generalized cross-matching with spatial average prior to threshold

The original version of cross-matching has a threshold (half-wave rectification) before spatial average (Equation 3), allowing us to derive the simple form of signal strength (Equation 5). However, the spatial average of binocular-interaction signals is likely to occur prior to thresholding nonlinearity in the visual system. In the threshold energy model (Lippert and Wagner, 2001), a threshold is applied to the outputs of energy models. Energy models produce signals that are already spatially averaged according to the receptive-field profiles (Ohzawa et al., 1990). Therefore, we also considered a more general form of cross-matching, defined as follows:

(6) $$G(d)=\langle {\left[\frac{1}{k}\sum {}_{\left(x,y\right)\in w}I_{L}\left(x,\;y\right)I_{R}\left(x-d,\;y\right)\right]}^{+}\rangle ,$$

in which half-wave rectification is applied after spatial average. We calculated the expected signal strength of the generalized cross-matching using the trinomial distribution (Equation 2). The distribution describes possible combinations of contrast-matched, contrast-reversed, and background pixels in the spatial window. The value of the generalized cross-matching is $\frac{1}{k}{\left[bn^{T}\right]}^{+}=\frac{{\left[n_{m}-n_{r}\right]}^{+}}{k}$ for a given ***n***. When the size of the spatial window (*k*) is 1, the expected value of the generalized cross-matching is equivalent to that of the original cross-matching (Equation 5). When the size of the spatial window is 2, the calculation using the trinomial distribution gives an expected signal strength (*S_G_*) with the following form:

(7) $$S_{G}\left(c,\rho |k=2\right)=\frac{\rho }{4}\left(\rho c^{2}+2c+\rho^{3}-3\rho +2\right).$$

When the size of the spatial window is infinite, the generalized cross-matching is equivalent to the half-wave-rectified cross-correlation: *G* (*d*|*k* = ∞) = [*C*(*d*)]^+^. Thus, the expected signal strength has the following form:

(8) $$\begin{array}{l} S_{G}\left(c,\rho |k=\infty \right)=G\left(d=d_{s}|k=\infty \right)\;-G\left(d\neq d_{s}|k=\infty \right)\\ \;={\left[C\left(d=d_{s}\right)\right]}^{+}-{\left[C\left(d\neq d_{s}\right)\right]}^{+}\\ \;={\left[c\rho \right]}^{+}. \end{array}$$

**Figure 6:**
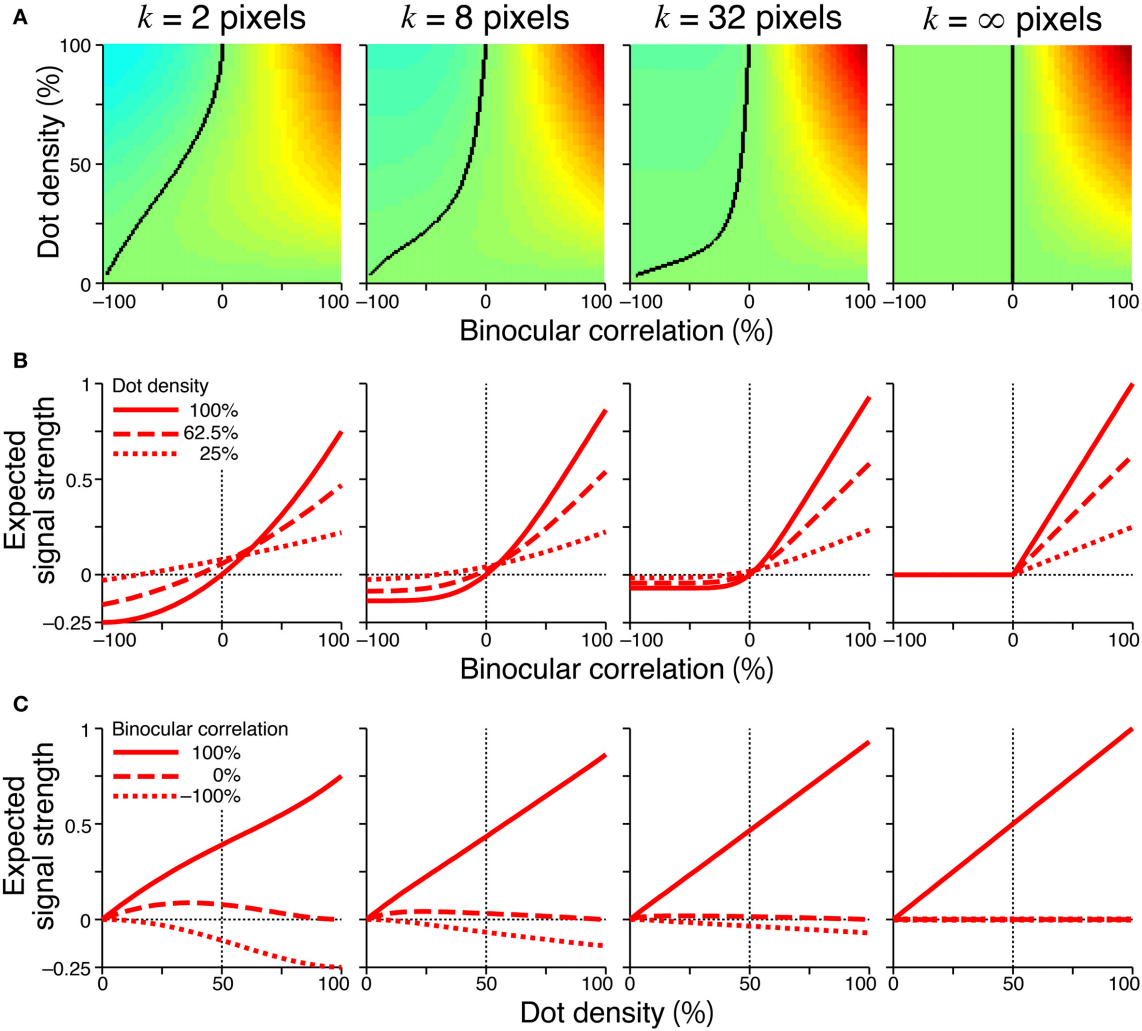
**Signal strength for generalized cross-matching**. Spatial averages were taken at various window sizes (2, 8, 32, and infinite pixels from the left to right columns) before thresholding nonlinearity. Other figure conventions are the same as those used in Figure 4, including the color scale of **(A)**, except for the y-axis range of **(B,C)**. In the rightmost panel of **(A)**, the solid black line indicates the boundary between positive and zero signal strengths, and the left part of the line is all zero.

Figure 6 showed the expected signal strength of generalized cross-matching for window sizes of 2, 8, 32, and infinite pixels. As the window size increased, the expected signal strength varied from that of the original cross-matching (Figure 4, right column) to the half-wave-rectified version of the cross-correlation (Figure 6, rightmost column). The negative signal near −100% correlation became weaker, whereas the positive signal near 100% correlation became stronger (Figure 6A). For the generalized cross-matching, the zero-signal contour was not a straight line (Figure 6A, black curve). For a larger window size, a larger part of the zero-signal contour stayed close to the vertical zero-correlation line, and the contour deflected more sharply at a lower dot density. For the 32-pixel window, the signal strength crossed zero at a near-zero correlation, even for 25% dot density (Figure 6B, middle right, dotted line). The signal strength was close to zero both for −100 and 0% correlations across the entire range of dot densities (Figure 6C, middle right, broken and dotted lines). However, original and generalized cross-matching shared some key characteristics up to a modest extent of spatial averaging (e.g., 8 pixels). In both cases, the signal strength crossed zero at larger correlations for larger dot densities (Figure 6B, middle left). The signal strength at −100% correlation decreased with dot density (Figure 6C, middle left, dotted line). We shall focus on these shared characteristics when we derive testable predictions regarding psychophysical performance in the next section.

### Simulation of the psychometric function in near/far discrimination

**Figure 7:**
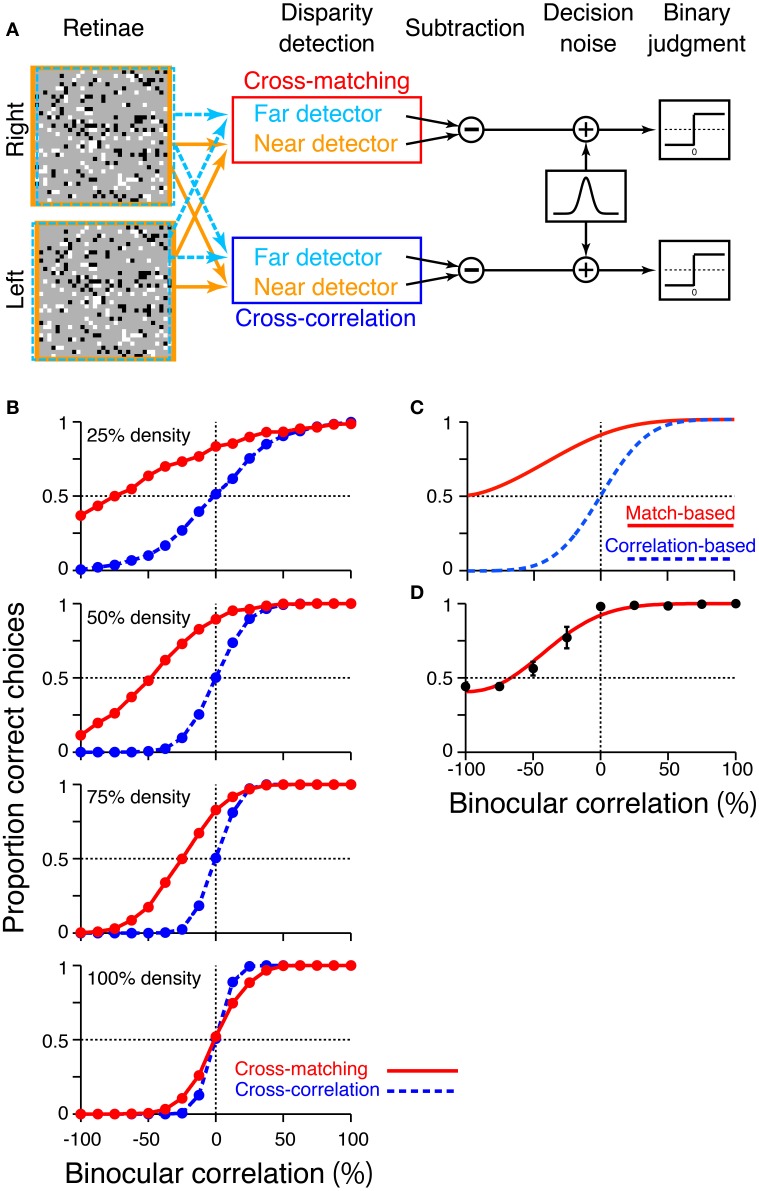
**Simulation of psychometric functions based on cross-correlation and cross-matching**. **(A)** Schematic diagram of the simulation. Near and far detectors were prepared for each computation (cross-correlation and cross-matching); their receptive fields are shown with orange and aquamarine squares on RDSs. The responses of these detectors were subtracted, corrupted with Gaussian noise, and rendered into a binary decision. **(B)** Simulated psychometric functions for a near/far discrimination task with low (top) to high (bottom) dot densities. **(C)** Ideal psychometric functions for the match-based (red) and correlation-based (blue) psychometric functions, hypothesized previously (adapted from Doi et al. (2011, 2013); the copyright of this figure belongs to the Association for Research in Vision and Ophthalmology). **(D)** Psychometric function observed in human subjects with 25% dot density and 0.03° disparity (Doi et al., 2011). Performance was averaged across four subjects. Error bars indicate standard error of means across subjects.

To examine how these disparity signals contribute to the psychophysical performance of depth judgment, we simulated near/far discrimination based on either cross-correlation (Equation 1) or cross-matching (the original definition; Equation 3). We generated RDSs using the same method we used for the simulation of signal-strength variability (Figure 5). The disparity of the central square was either 2 (uncrossed) or −2 (crossed) pixels. We prepared “near” and “far” detectors for each computation (cross-correlation and cross-matching). Response subtraction between these detectors was consistent with the standard decision-making mechanism in two-alternative forced-choice discriminations (Figure 7A; Shadlen et al., 1996; for physiological evidence in depth discrimination, see Uka and DeAngelis, 2004; Uka et al., 2005; Shiozaki et al., 2012). The response subtraction is also consistent with an “opponency” implemented in a generalized disparity energy model (Haefner and Cumming, 2008; Tanabe and Cumming, 2008). The near and far detectors had window (receptive-field) disparities corresponding to the crossed and uncrossed disparities of the RDSs, respectively (*d* = −2 and 2). The size and location of the window (*W* in Equations 1 and 3) were matched to those of the central square of an RDS.

We simulated a psychometric function by varying the correlation level from −100 to 100% for each of four dot densities (25, 50, 75, and 100%). In a given trial, we calculated the response of a detector averaged over 16 random-dot patterns. In our previous experiments, human subjects observed the same number of dot patterns on each trial (Doi et al., 2011). The model generated a near choice if the response of the near detector was larger than that of the far detector. The response was rendered into a binary choice after corruption with “decision noise” (Shadlen et al., 1996). The noise was sampled from a normal distribution with a mean of zero. The standard deviation of the normal distribution was 0.1 for the main simulations, whereas it was varied from 0.025 to 0.2 for supplementary simulations. We chose 0.1 for the main simulations so that the simulated psychometric function had a shape roughly similar to that of the observed psychometric function (Doi et al., 2011). However, the noise size does not affect the binocular correlation at which the psychometric function crosses the level of chance, a key prediction that we shall focus on later. Each single data point of the psychometric function was based on 1200 trials ([30 trials each for crossed and uncrossed disparity] × 20 blocks).

The psychometric functions had qualitatively different shapes for cross-correlation and cross-matching when the dot density was 25% (Figure 7B, top). The psychometric function of cross-matching was close to the chance level at −100% correlation, and performance gradually increased with increasing correlation. Notably, the performance was well above the chance level at 0% correlation (i.e., half-matched RDSs). By contrast, the psychometric function of the cross-correlation had an odd-symmetric shape that reflected its odd-symmetric signal strength (Figure 4B, left, dotted line). These simulated psychometric functions resembled the ideal match-based and correlation-based psychometric functions hypothesized previously (Figure 7C adapted from Doi et al., 2011, 2013). Under certain conditions, the near/far discrimination of human subjects agrees with the ideal match-based psychometric curve (Figure 7D; Doi et al., 2011). Thus, cross-matching serves as a simple computation underlying match-based depth perception.

Our previous psychophysical studies used only a dot density of 25%. Thus, the psychometric functions simulated with higher dot densities provide testable predictions regarding cross-matching. The performance at −100% correlation should monotonically decrease below the level of chance as the dot density is increased. The same prediction can be derived from the cross-correlation or pure energy models, because cross-correlation also produces a negative signal at −100% correlation, and the negative signal is stronger for higher densities (Figure 4C, left, dotted line). However, another prediction is specific to the cross-matching: the psychometric function crosses the level of chance, and the correlation level at this crossing point should be higher for larger densities (Figure 7B). These predictions are straightforward consequences of the signal strength (Figure 4B, right), and are preserved even when binocular signals are averaged modestly before threshold nonlinearity (Figure 6B, leftmost and middle left columns for 2-pixel and 8-pixel averages, respectively).

**Figure 8:**
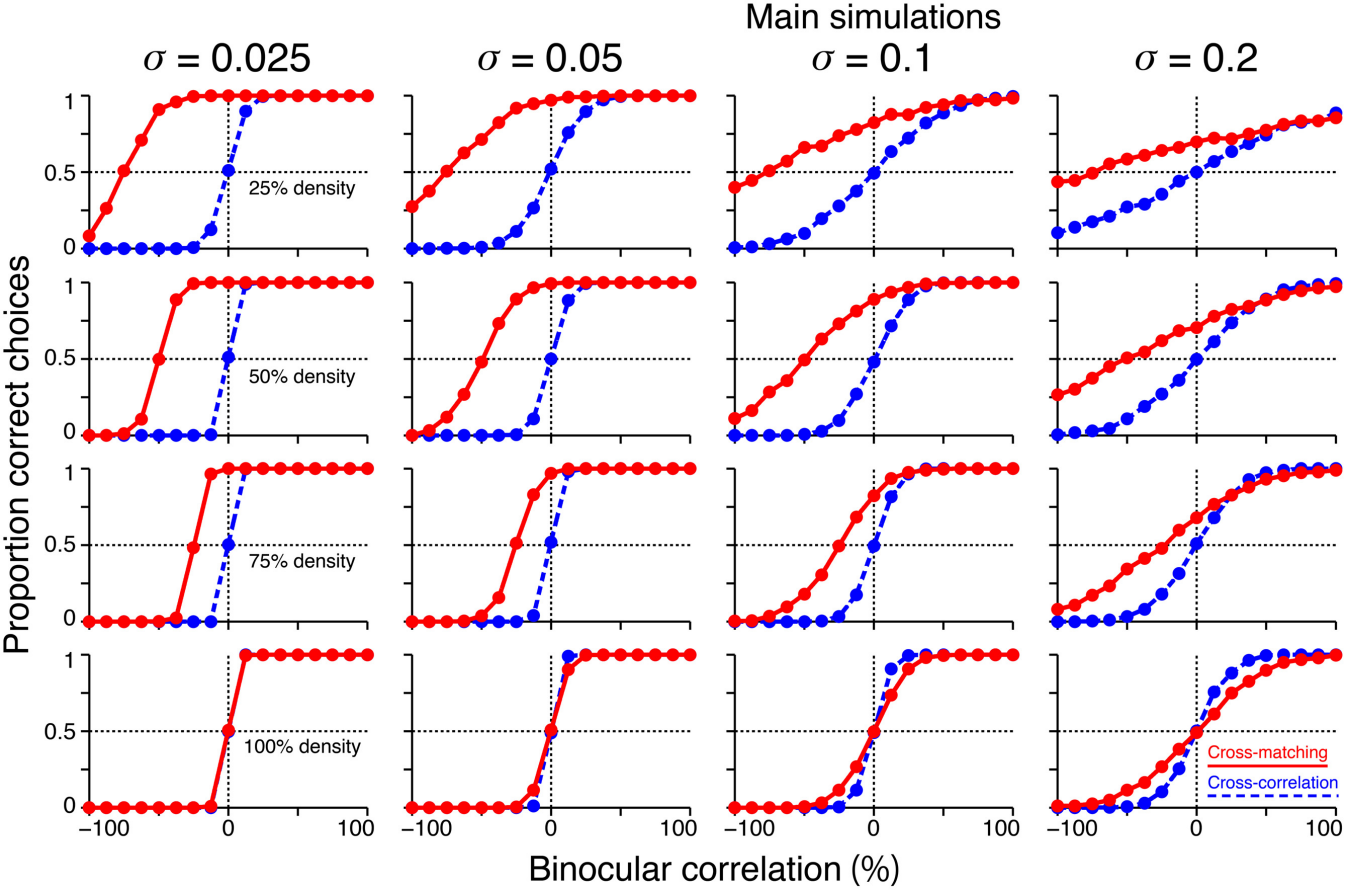
**Effects of noise size on the simulated psychometric functions**. Simulated psychometric functions for a near/far discrimination task with low **(top)** to high **(bottom)** dot densities and small **(left)** to large **(right)** decision noises. A decision-noise sigma of 0.1 (middle right column) was the value used in the main simulations (Figure 7B).

We performed additional simulations to examine how the size of the decision noise affects psychometric functions. First, for a given dot density, the psychometric function of the cross-matching crossed the chance level at the same binocular correlation, irrespective of the noise size (Figure 8, red). Thus, noise size does not affect our key prediction for the cross-matching: the binocular correlation for the crossing point should increase with dot density. Second, the simulated psychometric function of the cross-matching qualitatively agreed with the ideal match-based function (Figure 7C, red) and observed function (Figure 7D) at a dot density of 25% and a noise sigma of 0.1, but not at a higher density, irrespective of the noise size (Figure 8, red). As the density was increased, the performance at −100% correlation decreased away from the chance level. This discrepancy from the ideal and observed functions can be remedied by increasing the noise size. However, an increase in the noise size decreases the performance at 0% correlation toward the chance level, giving rise to another discrepancy. This is noteworthy because the actual dot density we used in our experiments was also 25%. The stereoscopic system may give rise to match-based depth perception (Figure 7C, red) for only low-density RDSs.

### Simulated psychometric function from threshold energy model

**Figure 9:**
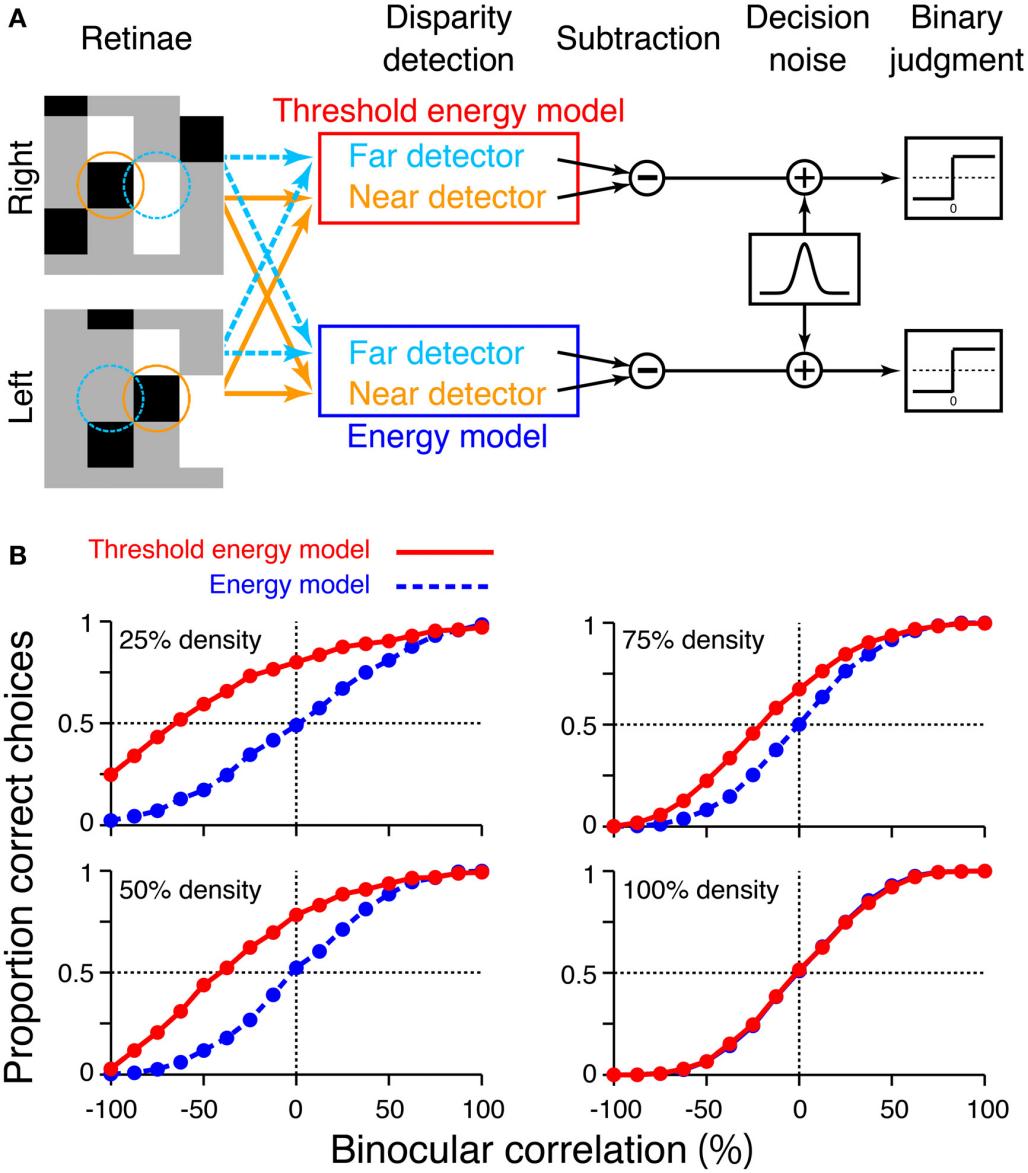
**Simulation of psychometric functions based on energy model and threshold energy model**. **(A)** Schematic diagram of the simulation. Orange and aquamarine circles indicate the receptive fields of the near and far detectors, respectively. The radius of the circle corresponds to two standard deviations of the receptive-field envelope. We simulated the response of the threshold energy model by passing the binocular-interaction component of the energy model response through half-wave rectification. **(B)** Simulated psychometric functions for a near/far discrimination task tested from low to high dot densities.

Finally, we attempted to confirm that the psychometric function simulated from the threshold energy model agrees with that from original cross-matching when the model's receptive field and stimulus dot have comparable sizes. To this end, we performed a similar simulation using the disparity detectors of a threshold energy model rather than those of cross-matching (Figure 9A). Likewise, we swapped the detectors of cross-correlation with those of a pure energy model. In our simulation, an energy-model detector consisted of two simple cells with different receptive-field phases (0 and 0.5π). For each simple cell, we calculated the inner product of the receptive field and stimulus for the left and right eyes (see the next paragraph for details). The results were summed across both eyes, followed by squaring nonlinearity, and integrated across the two simple cells to obtain an energy-model response. For the detectors of threshold energy model, we extracted the binocular-interaction component from the energy-model response by subtracting the monocular components (Tanabe et al., 2005). The binocular-interaction component was then passed through half-wave rectification (zero threshold). It is often useful to remove the monocular components from an energy-model response before output nonlinearity (Thomas et al., 2002). We integrated the frame-by-frame responses of detectors across 16 different random-dot patterns per trial and added Gaussian decision noise (mean, 0; standard deviation, 1000). We chose this value of standard deviation for the same reason as described above for the simulation for cross-matching. The near/far choice was obtained by comparing the responses between the near and far detectors, for both the energy model and the threshold energy model.

The receptive fields of the model simple cells were small two-dimensional Gabor functions, which we used previously (Equation 7 in Doi et al., 2013). Briefly, the vertical and horizontal widths of receptive field (the standard deviations of Gaussian envelope) were 0.05°, comparable to the receptive fields of some macaque V1 neurons (Prince et al., 2002). The far and near detectors had position disparities of 0.14° and −0.14°, respectively (i.e., even-symmetric tuning curve), and the RDSs also had stimulus disparities of ±0.14°. For simplicity, we matched these disparity magnitudes to a single-dot size used in our previous experiments (Doi et al., 2011, 2013). RDSs and receptive fields had a size of 24 pixels × 24 pixels; a single dot had a size of 8 pixels × 8 pixels; and one pixel corresponded to 0.0175°. Each single data point of the psychometric function was based on 1200 trials, as in the simulation of cross-matching.

We confirmed that the psychometric functions simulated from the threshold energy model behaved similarly to those simulated from original cross-matching (compare Figure 9B red and Figure 7B red). In particular, we observed the same characteristics in the shift of a psychometric curve as a function of dot density. As the dot density increased, the performance at −100% correlation decreased below the level of chance. Psychometric functions crossed the level of chance. The binocular correlation of this crossing point shifted toward larger correlations for larger densities. Thus, the predictions we made from original cross-matching held for the threshold energy model. The depth discrimination simulated from original cross-matching is qualitatively similar to that obtained from a threshold energy model, if the receptive-field size of the threshold energy model is comparable to the dot size of the RDS.

## Discussion

In this study, we proposed a nonlinear modification of cross-correlation, termed “cross-matching,” as the fundamental computation underlying threshold energy model. In cross-matching, binocularly combined signals undergo half-wave rectification before being spatially averaged. This threshold eliminates the negative signals from contrast-reversed combinations, while preserving the positive signals from contrast-matched combinations. We showed that cross-matching produced disparity tunings nearly insensitive to anticorrelated RDSs but sensitive to correlated and half-matched RDSs (Figure 3, right) when the dot density was low (e.g., 25%). As the dot density increased, the negative signal (i.e., inverted tuning) with anticorrelated RDSs became stronger (Figure 4C, right, dotted line), and the correlation level yielding a zero signal (i.e., flat tuning) became larger (Figure 4B, right). These characteristics were preserved up to a modest extent of pre-threshold spatial averaging (e.g., 8 pixels; Figure 6, middle left column). The psychometric curve simulated for near/far discrimination (Figure 7B, top, red) agreed with the previously hypothesized (Figure 7C) and observed (Figure 7D) match-based psychometric curves (Doi et al., 2011). Both simulated and observed results were obtained with 25% dot density. We derived testable predictions by increasing the dot density from 25 to 100%. The performance with anticorrelated RDSs gradually decreased below the level of chance with increasing dot density (Figure 7B, top to bottom). The correlation level yielding the chance performance also increased with dot density. A threshold energy model produced similar psychometric functions when the receptive-field size matched the dot size (Figure 9B). We suggest that cross-matching serves as a simple, abstract computation underlying the transformation of correlation-based disparity signals into match-based signals by the stereoscopic system.

### Limitation of cross-matching

We intended cross-matching to be parsimonious and minimally differentiated from cross-correlation. Given this, cross-matching is most useful under limited conditions. First, smooth disparity tuning can only be obtained after averaging signals across different dot patterns. Second, a uniform disparity plane should be embedded in an RDS. Third, the monocular windows (receptive fields) should have the same shape and location as the embedded depth plane. These conditions are often fulfilled when neuronal disparity tunings or psychometric functions are measured: mean firing rate or percent correct judgment is normally calculated as the average across different monocular dot patterns; a test plane often has a uniform disparity (but see Janssen et al., 2003); and the stimulus shape and location are tailored to match the receptive field of a neuron under physiological recording. In most psychophysical experiments, these stimulus parameters are fixed, and subjects can estimate the shape and location of the tested depth plane accurately in easy trials (e.g., correlated RDSs). Subsequently, the decision mechanism can monitor the neurons best suited to process the stimulus shape and location at hand.

Solving the correspondence problem in a more general situation may involve more sophisticated algorithms such as cooperative process (Marr and Poggio, 1976; Samonds et al., 2009), coarse-to-fine process (Marr and Poggio, 1979; Menz and Freeman, 2003; Chen and Qian, 2004), suppressive mechanisms (Tanabe et al., 2011; Tanabe and Cumming, 2014), and the detection of naturally impossible binocular inputs (Read and Cumming, 2007). Moreover, the integration of spatial frequency channels may underlie the reduced disparity selectivity and chance-level depth judgment of anticorrelated RDSs (Read and Eagle, 2000; Kumano et al., 2008; Hibbard et al., 2014). These mechanisms might fill the gap between the observed psychometric curve and the simulated curve based on cross-matching. The observed curves typically have sigmoidal shapes, even when the performance at −100% correlation is close to the level of chance (Figure 7D). By contrast, the psychometric curve of the cross-matching and that of the threshold energy model did not have a sigmoidal shape when the performance at −100% correlation was near the level of chance: the curves had positive, but not zero, slopes at −100% correlation for 25% dot density (Figure 7B, top, red; Figure 9B, top left, red). The sophisticated mechanisms described above might underlie the flattening of the observed psychometric function at the low correlation levels near −100%.

### Threshold energy model, generalized cross-matching, and cross-matching

Our generalized cross-matching is equivalent to cross-correlation followed by half-wave rectification. Binocularly multiplicative signals are spatially averaged within a window. The averaged signal, if negative, is nullified by a threshold. These processes are directly related to those of a threshold energy model described below. First, disparity energy models compute the spatial average of monocular images (i.e., weighted sum within monocular receptive fields) and encode the binocular interaction through binocular summation and squaring nonlinearity. Second, this spatially averaged binocular signal is nullified by a threshold if the net binocular interaction is negative.

The threshold added to energy model explains the reduced disparity selectivity for anticorrelated RDSs (Lippert and Wagner, 2001; Nieder and Wagner, 2001). This is a simple solution for tuned-excitatory cells, because the disparity-dependent modulation for anticorrelated RDSs occurs below the baseline response level for these cells. Further combination of these subunits expains the reduced selectivity for odd-symmetric tuning curves (Haefner and Cumming, 2008; Tanabe and Cumming, 2008).

The original version of cross-matching is a special case of generalized cross-matching in which the spatial window has a size of one pixel; thus, the threshold operates on binocularly multiplicative signals with single-pixel resolution. However, some characteristics of original cross-matching are preserved even when a modest amount of spatial averaging (e.g., 8 pixels) occurs prior to the threshold. The signal strength at −100% correlation decreased with dot density (Figure 6C, middle left, dotted line), and the correlation level yielding a zero signal increased with dot density (Figure 6B, middle left). Under certain conditions, original cross-matching is a reasonable simplification of a threshold energy model, with the key being the relationship between the size of a stimulus dot and the size of the spatial receptive field: when the two sizes match, original cross-matching captures the essential characteristics of the threshold energy model. The psychometric functions simulated from original cross-matching and the threshold energy model shifted in a similar manner depending on the dot density (compare Figure 7B and Figure 9B).

### Psychophysical considerations of the extent of spatial averaging prior to threshold

In match-based depth perception, the extent of the pre-threshold spatial window is likely to be small, not significantly larger than a few dots. We used a dot size of 0.14° × 0.14° (Doi et al., 2011, 2013); some disparity-selective neurons in macaque V1 have receptive fields as small as this (estimated from disparity tuning width in Prince et al., 2002). Neurons with small receptive fields preferentially encode fine disparities (size-disparity correlation; Prince et al., 2002). Indeed, depth perception is more strongly match-based, as opposed to correlation-based, with finer disparities (Doi et al., 2011). Furthermore, three experiments have provided evidence for a fine spatial window underlying match-based depth perception. First, match-based depth perception is stronger at smaller eccentricities, where receptive-field sizes are smaller (Figure 11 of Doi et al., 2013). Second, match-based depth perception is stronger for slower pattern refreshes (Doi et al., 2013). Neurons preferring slow temporal inputs tend to prefer fine spatial features (DeAngelis et al., 1993). Third, match-based depth perception relies on the binocular correlation calculated within a small area (Doi et al., 2013). Depth perception for a half-matched RDS degrades when a contrast-matched dot is placed right next to every contrast-reversed dot in the RDS. The distance between the contrast-matched and contrast-reversed dots was 0.15°. These results suggest that spatial averaging takes place within a small area prior to threshold nonlinearity (generalized cross-matching with a small window). Overall, generalized cross-matching is the fundamental computation underlying the threshold energy model and match-based depth perception. Original cross-matching is a reasonable simplification, if experimental manipulation does not change local correlation statistics within the receptive field.

### Advantage of original over generalized cross-matching

Simplicity is the advantage of original over generalized cross-matching. The signal strength of original cross-matching is expressed as a simple function of binocular correlation and dot density (Equation 5). By contrast, it is not clear whether the signal strength of generalized cross-matching can be expressed as a simple function of correlation, density, and spatial-window size.

By taking the advantage of its simplicity, we showed that original cross-matching is equivalent to a model in which the half-wave rectification is placed immediately after monocular contrast signals. Equation 3 for original cross-matching can be rewritten as:

(9) $$\begin{array}{l} M\left(d\right)=\langle \frac{1}{k}\sum {}_{\left(x,y\right)\in W}\{{\left[I_{L}\left(x,y\right)\right]}^{+}{\left[I_{R}\left(x-d,y\right)\right]}^{+}\\ \;+{\left[-I_{L}\left(x,y\right)\right]}^{+}{\left[-I_{R}\left(x-d,y\right)\right]}^{+}\}\rangle , \end{array}$$

where *I_L_* and *I_R_* can be considered as the outputs of ON monocular channels and −*I_L_* and −*I_R_* as the outputs of OFF monocular channels. The binocular multiplication is taken separately for the thresholded outputs of the ON and OFF channels. This expression invokes another modified energy model that was also developed to explain the reduced disparity selectivity for anticorrelated RDSs (Read et al., 2002). In this model, threshold nonlinearity is placed on the outputs of monocular simple cells. The model has four monocular channels differing in receptive-field phase. The model combines the thresholded outputs of the monocular simple cells across the two eyes separately for each channel. The encoded binocular interactions are integrated across channels to obtain the final output. These steps are similar to those expressed in Equation 7. The equivalence of Equations 3 and 7 suggests the equivalence of the two modified energy models with thresholds inserted at different steps: one after monocular cells (Read et al., 2002), and the other after a binocular energy cell (Lippert and Wagner, 2001; see also Read et al., 2002 for the proof). However, we should note that the model of Read et al. (2002) has an advantage over that of Lippert and Wagner (2001), in that the reduced selectivity of an odd-symmetric tuning curve can be realized in the same mathematical framework.

### Threshold nonlinearity and peak detection

In our simulations, cross-correlation and disparity-energy model always produced reversed depth perception for anticorrelated RDSs, because we adopted the opponency (i.e., response subtraction) between near and far units as a mechanism to transform sensory responses into near/far decisions. The opponency mechanism is widely supported by theoretical and physiological studies in motion and stereoscopic depth perception (Shadlen et al., 1996; Prince and Eagle, 2000; Ditterich et al., 2003; Uka and DeAngelis, 2004; Uka et al., 2005; Shiozaki et al., 2012). However, peak detection (winner take all or maximum operation) is another influential mechanism implicated in the chance-level performance for anticorrelated RDSs (Read and Eagle, 2000; Hibbard et al., 2014). The cross-correlation function does not have a positive peak for anticorrelated RDSs (Figure 3B, blue), and neither does a population activity of energy model neurons, if averaged across spatial frequency channels (Hibbard et al., 2014). Thus, peak detection can only produce the chance-level performance from cross-correlation or disparity-energy model for anticorrelated RDSs.

The threshold nonlinearity used in cross-matching and threshold energy model, in effect, implements a peak detection in the framework of opponency decoding. If the dot density is low, the threshold nonlinearity eliminates the response dip present in sensory responses (compare Figure 3B red and blue), so that only the positive response peak contributes to near/far decisions, if any. Our experiments show that the performance of human observers for low-density, anticorrelated RDSs varies from the chance level to the reversed depth perception depending on the magnitude of disparity and the refresh rate of the dot pattern (Tanabe et al., 2008; Doi et al., 2011, 2013). Although the performance varies, all stereoscopic processes originate in the primary visual cortex, which computes disparity energy (Ohzawa et al., 1990; Cumming and Parker, 1997). The presence or absence of threshold nonlinearity after V1 may cause the variability in performance from the common disparity signals in V1. Without the threshold nonlinearity, the signals are fed into the opponency decision mechanism to give rise to the reversed depth perception for anticorrelated RDSs. With the nonlinearity, the response dip is eliminated, and the disparity representation devoid of any positive peak results in chance-level performance.

## Conclusion

Cross-correlation is the fundamental computation underlying the disparity selectivity of energy models. We proposed a modified cross-correlation, termed “cross-matching,” as an equivalent computation for the energy model followed by threshold nonlinearity (Lippert and Wagner, 2001). Cross-matching can explain the reduced disparity selectivity for anticorrelated RDSs, as well as the disparity selectivity for half-matched RDSs (half-and-half mixtures of correlated and anticorrelated RDSs). The simulated psychometric function agreed with the ideal match-based psychometric function hypothesized and observed previously (Doi et al., 2011), and also agreed with the psychometric function simulated with a threshold energy model. In original cross-matching, threshold nonlinearity operates on binocular signals with single-pixel resolution. Some characteristics of original cross-matching were preserved even when a modest amount of spatial averaging (e.g., 8 pixels) occurred prior to the threshold. We suggest that cross-matching serves as a minimal computation underlying match-based disparity representation (Nieder and Wagner, 2001; Janssen et al., 2003; Tanabe et al., 2004; Kumano et al., 2008) and depth perception (Doi et al., 2011), just as cross-correlation serves as the fundamental computation underlying the initial cortical representation of disparity (Ohzawa et al., 1990; Fleet et al., 1996; Qian and Zhu, 1997; Anzai et al., 1999) and some aspects of depth perception (Cormack et al., 1991; Filippini and Banks, 2009; Doi et al., 2011, 2013).

### Conflict of interest statement

The authors declare that the research was conducted in the absence of any commercial or financial relationships that could be construed as a potential conflict of interest.
